# A Prescription for Achieving Equitable Access to Antiobesity Medications

**DOI:** 10.1001/jamahealthforum.2023.0493

**Published:** 2023-04-07

**Authors:** Davene R. Wright, Jingchuan Guo, Inmaculada Hernandez

**Affiliations:** Department of Population Medicine, Harvard Medical School and Harvard Pilgrim Health Care Institute, Boston, Massachusetts; Department of Pharmaceutical Outcomes and Policy, College of Pharmacy, University of Florida, Gainesville; Division of Clinical Pharmacy, Skaggs School of Pharmacy and Pharmaceutical Sciences, University of California, San Diego (UCSD)

Obesity is a risk factor for cardiovascular disease, type 2 diabetes (T2D), and premature mortality. In the US, approximately 36% of adults have obesity; however, large disparities exist across racial, ethnic, and income groups.^1^ Approximately 48% of non-Hispanic Black and 43% of Hispanic adults have obesity vs 34% of non-Hispanic White adults.^1^ Nearly 40% of adults with household incomes below 350% of the federal poverty level have obesity, vs 31% of those above 350% of the federal poverty level.^1^

Obesity has multifaceted causes and can be addressed by numerous clinical and policy approaches, including behavioral, surgical, and pharmacological interventions. However, behavioral interventions and bariatric surgery are limited by their modest population-level effectiveness,^2^ particularly among groups disproportionately affected by obesity.^2,3^

## The Promise of New Antiobesity Medications (AOMs)

The US Food and Drug Administration (FDA) has approved glucagon-like peptide-1 (GLP-1) receptor agonists liraglutide (December 2014) and semaglutide (June 2021) as pharmacologic treatments for obesity.^2^ These drugs were previously approved for T2D and used off-label for obesity. Additionally, the FDA is expected to soon review the gastric inhibitory polypeptide and GLP-1 receptor agonist tirzepatide.^2^ These newer AOMs have favorable safety profiles and demonstrate 14.9% to 20.9% weight loss over 12 months vs 6.9% to 10% for older AOMs including orlistat, phentermine/topiramate, and naltrexone/bupropion.^2,4^

Newer AOMs are indicated for individuals with a body mass index (BMI) of more than 27 (calculated as weight in kilograms divided by height in meters squared) and 1 obesity-related comorbidity (eg, hypertension) or individuals with a BMI above 30.^2^ More than 142 million (40%) US adults meet these indications.^2^ Newer AOMs are not deemed cost-effective at their current prices of approximately $1000 per month, and would have to take 40% to 60% price reductions to meet commonly accepted willingness-to-pay thresholds in cost-effectiveness analyses.^2^ The disproportionate burden of obesity on underserved populations, the sheer number of eligible patients, the high costs of new AOMs, and the lack of coverage under public health plans raise concerns around inequities in AOM access, which could exacerbate health disparities.^1^

## A Multistakeholder Prescription for Improving Equity in AOM Access

Pharmaceutical manufacturers, health care payers, prescribers, and researchers can play crucial roles to ensure that the uptake of newer AOMs does not exacerbate existing inequities in obesity, T2D, cardiovascular disease, and other obesity-related outcomes. To that end, we offer the following prescriptions for each of these key stakeholders.

### Pharmaceutical Manufacturers

High AOM costs create major access barriers, particularly for uninsured patients, patients enrolled in high-deductible health plans, and those in insurance plans that do not cover AOMs. For patients with AOM insurance coverage, high costs prompt payers to establish administrative barriers, such as prior authorization and step therapy that ultimately delay patient access and contribute to nonadherence. Manufacturers have the ability to improve AOM access and promote equity by reducing prices to ranges within common societal willingness-to-pay thresholds.^2,5^

Manufacturers also have a responsibility to ensure an adequate medication supply. Within a year of the approval of semaglutide for obesity, shortages arose due to increased demand and inadequate supply, which also affected the availability of semaglutide for T2D patients.

### Health Care Payers

Currently, the Medicare Part D statute excludes weight-loss drugs from coverage. Medicaid state programs can opt to exclude weight-loss drugs from coverage, and only a minority of Medicaid programs cover obesity medications.^6^ Policy efforts to amend the Medicare Part D statute to allow coverage of AOMs are ongoing,^7^ but these reforms would not affect Medicaid.

Private payers and Medicaid programs should consider patient-centered frameworks for AOM coverage and formulary placement decisions that account for downstream AOM benefits associated with the prevention of obesity-related comorbidities. They should also consider the burden that utilization management tools place on patients with limited health insurance literacy. This concern is particularly relevant for Medicaid and low-cost Marketplace plans that cover disadvantaged individuals, as the advancement of health equity requires improved medication access among those with greater unmet need, as opposed to an increased uptake among groups that already have access.

All payers should consider forward-looking coverage decisions that account for downstream long-term AOM benefits associated with preventing obesity-related comorbidities and complications over realistic time horizons, as captured by cost-effectiveness models.^2^ Coverage decisions should be revisited as other viable treatments arise and as longer-term data on AOM effectiveness and safety become available.

### Prescribers

As the therapeutic arsenal for obesity expands, heterogeneity in preferences for treatment will increase. Prescriber decision support can align treatment recommendations with patient preferences to improve adherence and mitigate clinicians’ implicit and explicit biases.^8^ Decision support development should be informed by qualitative and quantitative data on patients’ preferences regarding AOM demand (which may vary by health status or demographic subgroup) and regarding expectations for how AOMs will affect patient health. Prescriber-facing real-time benefit tools, which report patients’ out-of-pocket costs and insurance-based coverage restrictions for proposed treatments within the electronic health record, can also address financial and administrative barriers in prescribing AOMs.^9^ Finally, prescriber training is recommended to combat biases against patients who are obese or from underserved racial and ethnic groups, which may influence the prescribing of AOMs.

### Health Care Researchers

Clinical trials often fail to generate evidence on the long-term effects of AOMs on obesity-related comorbidities and mortality outcomes. Robust observational studies and economic evaluations of AOMs are needed to establish their postmarketing effectiveness and safety profile, quantify their value, and measure equity in access. Three approaches could address this need.

First, observational studies should describe the long-term effectiveness of AOMs and test for potential treatment heterogeneity. Such studies are critical because of the behavioral component of weight loss, which can be subject to Hawthorne effects in clinical trials. In planning these analyses, researchers must overcome barriers regarding the limited availability of data sources linking objectively measured weight and BMI with claims data to track longitudinal exposures and outcomes.

Second, the evaluation of actual equity in AOM use and the identification of factors that underlie inequities in AOM use are crucial steps to identify targets for interventions that improve equity. This research requires robust data on socioeconomic status, race, ethnicity, and place of residence to support studies that evaluate the intersection of social determinants of health with prescribing and use of AOMs. Electronic health record and data vendors can demonstrate a commitment to equity by developing mechanisms that facilitate access to these variables. Public and private data vendors should develop protocols that enable investigators to observe these data with high granularity while preserving privacy and security standards.

Lastly, cost-effectiveness analyses are needed to inform payer coverage decisions.^2,5^ Researchers should consider the appropriate choice of comparator(s)for cost-effectiveness analyses, as obesity interventions focused on lifestyle modifications may be unrealistic comparators due to their lack of accessibility and scalability. A no-intervention-or-support option (ie, placebo) may be the most realistic treatment alternative for AOM-related cost-effectiveness analyses.^5^ These AOM economic evaluations should also explicitly consider equity; distributional cost-effectiveness and multicriteria decision analysis methods can incorporate equity into decision-making processes.^5,10^ Finally, value of information analyses conducted alongside model-based cost-effectiveness analyses can quantify the cost of payers delaying coverage decisions and can prioritize high-value research targets to reduce decision uncertainty.^5^

## Conclusions

Newer AOMs hold promise, but uneven access to these medications could exacerbate obesity disparities. Health care stakeholders have major responsibilities to ensure equitable uptake of AOMs for the millions of individuals affected by obesity.
